# Disseminated mpox with suspected immune reconstitution inflammatory syndrome in a person living with HIV: A case report from South Kivu, Democratic Republic of the Congo

**DOI:** 10.1016/j.idcr.2026.e02733

**Published:** 2026-08-19

**Authors:** Alain Balola Ntaboba, Christian Tshongo, Guillaume Ashuza Shamamba, Victoire Urbain Hatu’m, Alfred Kesheni Bisimwa, Igor Akilimali Batumike, Samuel Lwamushi Makali, David Lupande Mwenebitu, Joanne Byrne, Virginie Gautier, Eoin R. Feeney, Jane A. O’Halloran, Patrick W.G. Mallon, Robert Colebunders, Patrick D.M.C. Katoto

**Affiliations:** a Center for Tropical Diseases and Global Health, Faculty of Medicine, Université Catholique de Bukavu, Bukavu, Democratic Republic of the Congo; b Centre for Experimental Pathogen Host Research, University College Dublin, Dublin, Ireland; c Global Health Institute, University of Antwerp, Antwerp, Belgium; d Hôpital Provincial Général de Référence de Bukavu, Université Catholique de Bukavu, Bukavu, Democratic Republic of the Congo; e European Society of Clinical Microbiology and Infectious Diseases (ESCMID), Basel, Switzerland; f Centre de Recherche en Sciences Naturelles de Lwiro, Lwiro, Democratic Republic of the Congo; g Department of Infectious Diseases, St Vincent’s University Hospital, Elm Park, Dublin, 4, Ireland; h Centre for General Medicine and Global Health, University of Cape Town, South Africa; i Cochrane South Africa, South African Medical Research Council, Cape Town, South Africa

**Keywords:** Mpox, HIV, Antiretroviral therapy, Immune reconstitution inflammatory syndrome, Democratic Republic of the Congo, Case report

## Abstract

**Background:**

Severe mpox is increasingly reported in people living with HIV (PLHIV). While immune reconstitution inflammatory syndrome (IRIS) is well-characterised in other opportunistic infections, its role in the acute clinical course of mpox remains poorly understood.

**Case presentation:**

A 42-year-old man from eastern South Kivu, Democratic Republic of the Congo (DRC), with newly diagnosed HIV initiated tenofovir/lamivudine/dolutegravir. Two weeks later, he developed severe disseminated clade I mpox, confirmed by PCR, with more than 250 polymorphic lesions. Around day 7 of admission, while afebrile, he experienced paradoxical clinical worsening with confluent hemorrhagic and necrotic plaques, which were highly suggestive of mpox-associated IRIS. A pragmatic management protocol consisting of a 6-week tapered course of oral prednisone, broad-spectrum oral antibiotics (levofloxacin and clindamycin), high-dose acyclovir, and supportive care led to progressive skin healing and clinical recovery while ART was continued. He was discharged after 62 days at the family's request. Unfortunately, 3.5 weeks post-discharge, he died at home following the application of traditional topical preparations to residual lesions.

**Conclusions:**

This case highlights the need to consider mpox-associated IRIS in the differential diagnosis of unexplained clinical worsening of mpox in PLHIV who recently initiated ART. Although definitive confirmation of IRIS was not possible because of limited immunological investigations, the temporal association with ART initiation and the clinical course were consistent with this diagnosis. This case underscores the need for standardized diagnostic criteria, prospective evaluation of adjunctive anti-inflammatory therapies, and strengthened post-discharge community follow-up in resource-limited settings.

## Background

The re-emergence of mpox, caused by the monkeypox virus (MPXV), has evolved into a protracted global public health emergency, with distinct epidemiological and clinical paradigms emerging across different geographic clades [1]. As of 17 May 2026, Africa reported 68,267 confirmed mpox cases and 294 deaths across 36 countries [1]. The Democratic Republic of the Congo (DRC) remains the primary endemic reservoir, with clade Ib MPXV driving sustained and severe transmission dynamics in South Kivu [1].

While mpox is usually self-limiting within 3–4 weeks in immunocompetent individuals, vulnerable hosts, particularly those living with advanced immunosuppression, face disproportionately severe, protracted, and disseminated infections. The U.S. Centers for Disease Control and Prevention defines severe mpox by multiple large confluent lesions, necrotic lymphadenopathy, or involvement of sensitive areas [2], which contribute to morbidity and mortality among PLHIV and the elderly [3,4]. A systematic review reported a 56.6% increased risk of hospitalisation among PLHIV with mpox compared with HIV-negative individuals (95% CI 18–107.7%), suggesting a substantially higher disease burden in this population [5]. Among PLHIV, advanced HIV disease is responsible for poor outcomes as it impairs cellular immunity through low CD4^+^ T-cell counts or high viral loads, enhancing susceptibility to disseminated mpox and complications [[6], [7], [8]].

In PLHIV presenting with opportunistic infections, clinical worsening after initiating antiretroviral therapy (ART) can signal immune reconstitution inflammatory syndrome (IRIS) [9]. IRIS manifests either as "paradoxical" IRIS (worsening of a treated infection after starting ART) or "unmasking" IRIS (flare-up of an underlying, undiagnosed infection) [10]. Both types involve an exaggerated inflammatory response during immune recovery despite virologic improvement. Although well-characterized for tuberculosis [11], cryptococcosis [12], and Kaposi’s sarcoma, mpox-associated IRIS remains poorly understood, and studies show no clear excess of IRIS-like events [13,14], underscoring the value of detailed individual case reports.

Here, we report a fatal case of disseminated clade I mpox with features suggestive of ART-associated IRIS from South Kivu, eastern Democratic Republic of the Congo.

## Case presentation

### Patient and history

A 42-year-old previously healthy heterosexual man, married and residing in a rural community in the Miti-Murhesa health zone, South Kivu province, eastern DRC, was admitted to the LWIRO Mpox Treatment Center. He had been diagnosed with HIV infection approximately one month earlier after presenting with progressive involuntary weight loss and chronic diarrhoea, which prompted HIV testing. He subsequently initiated a fixed-dose combination of tenofovir 300 mg, lamivudine 300 mg, and dolutegravir 50 mg once daily approximately two weeks before the onset of cutaneous symptoms. The patient reported no history of animal or bushmeat contact, or documented close household contact with individuals presenting with acute skin rashes. Detailed sexual history was not exhaustively addressed due to social stigma and cultural sensitivities in the rural setting; however, there were no secondary cases identified among his close household members or neighbours. He reported no prior history of recurrent opportunistic infections, tuberculosis, or hospitalisation. The patient reported involuntary weight loss in the month preceding his HIV diagnosis and admitted to self-treatment with traditional herbal preparations, with only transient symptomatic relief.

### Symptom onset and clinical course before admission

Approximately two weeks after starting ART, the patient developed progressive fever associated with localised pruritus, initially limited to the upper limbs, chest and abdomen, which extended over the following days to the lower limbs, face and extremities, with multiple scratch-related secondary lesions. These cutaneous manifestations were preceded by a prodrome of headache, myalgia, malaise, anorexia and arthralgia. Over the next two weeks, his condition worsened with recurrence of fever, generalisation of the rash into vesiculo-pustular lesions, feeding difficulties, and progressive deterioration of his general status, prompting admission to the LWIRO Mpox Treatment Center.

### Findings on admission

**Fig. 1:**
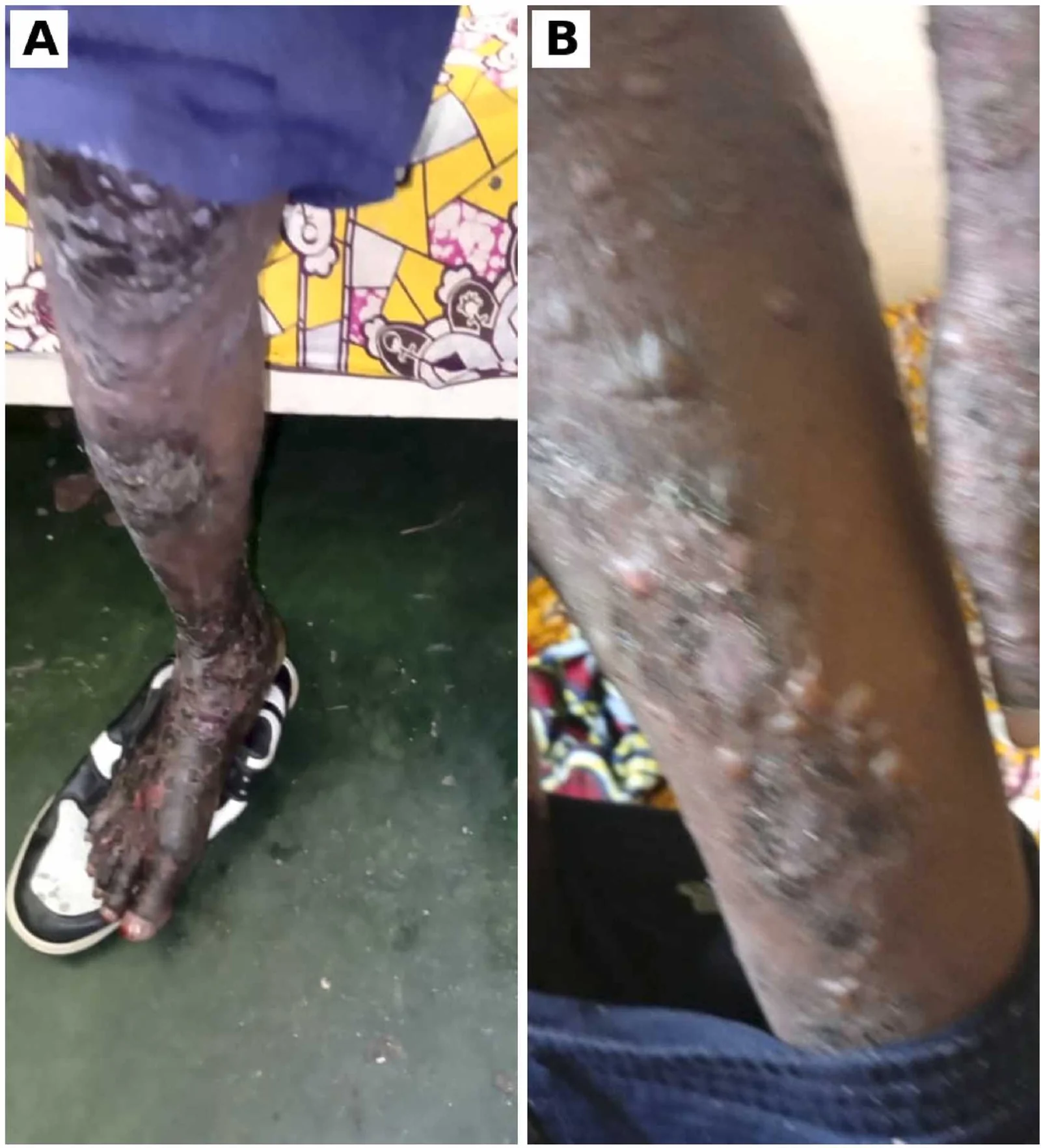
Disseminated polymorphic cutaneous lesions on admission to the LWIRO Mpox Treatment Center. (A) Right lower limb showing extensive ulcero-crusted plaques with necrotic centres and confluent crusts on the leg and foot. (B) Left forearm showing multiple vesiculo-pustular and umbilicated lesions on a background of erythematous skin. Lesions are at different stages of evolution, a hallmark of mpox in this patient.

On admission (Day 1), the patient appeared chronically ill but conscious, with marked asthenia, and clinical signs of dehydration and a body weight of 57 kg. Vital signs revealed a fever (body temperature of 38.5 °C), tachycardia with a heart rate of 108 beats/min, hypotension with a blood pressure of 97/54 mmHg, and an oxygen saturation of 96% on ambient air. No clinical features suggestive of neurological, respiratory, or gastrointestinal involvement were identified. Mucosal involvement was confined to lesions in the oral cavity (including oral candidiasis) and external genitalia. Dermatological examination revealed over 250 disseminated polymorphic skin lesions involving the face, oral cavity, chest, abdomen, back, limbs, hands and feet. These baseline lesions consisted predominantly of discrete, well-circumscribed papules, vesicles, vesiculo-pustular lesions, and isolated ulcerated crusts without widespread confluent haemorrhagic or necrotic plaques (Fig. 1). PCR testing of swab exudates confirmed MPXV Clade I. Repeat HIV serology was reactive and plasma HIV−1 RNA viral load was 688 copies/m. Due to regional laboratory constraints at the rural treatment centre, baseline CD4+ T-cell counts, STI co-infection screening (e.g. syphilis serology, HSV PCR) could not be performed.

Basic laboratory investigations showed a total leukocyte count of 4750 cells/µL (2480 neutrophils/µL, 1940 lymphocytes/µL and 180 monocytes/µL), haemoglobin of 11 g/dL with a mean corpuscular volume of 88.6 fL and a haematocrit of 33.2%; a platelet count of 323,000/µL; and glycated haemoglobin (HbA1c) of 5.2%. These findings indicated mild normocytic anaemia without leukocytosis or thrombocytopenia. Based on the clinical presentation, a diagnosis of severe disseminated mpox in the context of HIV infection was made. Culture of pus obtained from cutaneous lesions yielded heavy growth of multidrug-resistant *Escherichia coli*, susceptible to imipenem, meropenem, levofloxacin, trimethoprim-sulfamethoxazole, ciprofloxacin, and chloramphenicol.

### Initial management and in-hospital evolution

A working diagnosis of severe disseminated mpox complicated by secondary bacterial cutaneous sepsis was established. Empirical antibiotic therapy was initiated with intravenous ceftriaxone (1 g twice daily), oral cloxacillin (500 mg three times daily), and intramuscular gentamicin (160 mg once daily, discontinued on day 5). Adjunctive therapy included oral nystatin (500,000 IU twice daily) for oral candidiasis, tramadol (50 mg twice daily) for analgesia, and intravenous rehydration with 5% dextrose and Ringer’s lactate. Daily local wound care and psychosocial support were provided. ART with TLD was continued throughout admission. MPXV-specific therapies such as tecovirimat and brincidofovir were unavailable at the treatment centre.

**Fig. 2:**
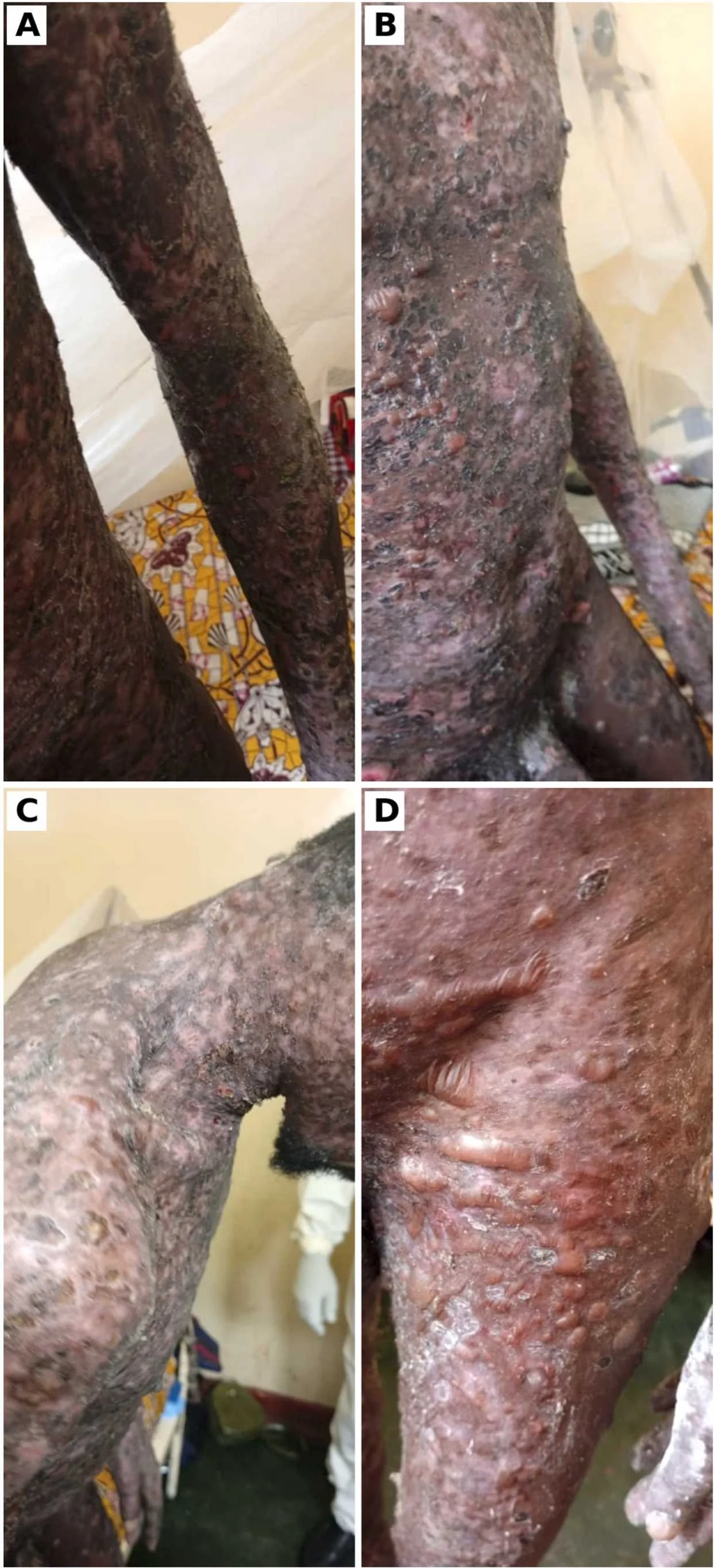
Paradoxical clinical worsening on days 7–10 of hospitalisation, potentially as a consequence of mpox-associated immune reconstitution inflammatory syndrome (IRIS). (A) Lower limbs showing extensive, confluent dark-purplish haemorrhagic-appearing crusted plaques covering virtually the entire skin surface. (B) Right flank and abdomen with confluent vesiculo-pustular and necrotic lesions and haemorrhagic crusts. (C) Posterior trunk and right shoulder showing widespread coalescence of lesions into large purple-red plaques with central necrosis. (D) Close-up of the medial thigh showing dense, polymorphic pustules and bullae in different stages of evolution, with serous and hemorrhagic exudation. These findings developed despite ongoing antiretroviral therapy, broad-spectrum antibiotics and supportive care, while the patient was no longer febrile, prompting initiation of the structured oral IRIS protocol summarised in Table 1.

No significant clinical improvement occurred during the first week. Around day 7, despite the resolution of fever, the patient experienced a paradoxical worsening of his cutaneous and systemic features. New pustular and bullous lesions appeared in previously unaffected areas, and existing lesions coalesced into large haemorrhagic, necrotic, and ulcerated plaques predominantly on the trunk, limbs, and back (Fig. 2). This clinical decline was accompanied by increased pain, severe dysphagia, and deterioration in his general condition. The absence of fever at this stage argued against uncontrolled systemic bacterial sepsis as the primary driver of this clinical deterioration.

### Management of the suspected mpox-associated IRIS

Given (i) the clear temporal relationship between recent ART initiation (~2 weeks before symptom onset, ~4 weeks before paradoxical worsening); (ii) the low admission HIV−1 RNA viral load (688 copies/mL), potentially reflecting an early virologic response to the dolutegravir-based regimen; (iii) the absence of fever during the worsening phase, which made ongoing systemic bacterial sepsis less likely; (iv) the polymorphism of the lesions with potential additional contribution of HSV/VZV co-infection (although not laboratory confirmed), as frequently described in PLHIV with anogenital involvement; and (v) the incompatibility of the lesion morphology with severe drug eruptions (Stevens–Johnson syndrome/toxic epidermal necrolysis), a multidisciplinary case discussion concluded that the most likely diagnosis was mpox-associated IRIS in a PLHIV who had recently initiated ART.

The management protocol introduced (Table 1) combined continued ART with a tapering course of oral prednisone to target hyperinflammation. Due to a loss of intravenous access from extensive limb lesions, antimicrobial coverage transitioned to oral therapies. High-dose oral acyclovir (800 mg five times daily for 7–10 days was incorporated empirically into the pragmatic treatment regimen even though it possesses no activity against MPXV. This introduction was driven by the strong clinical suspicion of an underlying herpes simplex virus (HSV−1/2) or varicella-zoster virus (VZV) co-infection—frequently co-occurring in severely immunosuppressed PLHIV presenting with atypical, severe, or necrotic mucosal and anogenital lesions—which could not be definitively ruled out by molecular diagnostics available on site.

**Table 1:** Clinical management of the patient with suspected mpox-associated immune reconstitution inflammatory syndrome.

**Component**	**Intervention and rationale**
Antiretroviral therapy	Continuation of TLD (tenofovir 300 mg/lamivudine 300 mg/dolutegravir 50 mg) once daily, under direct supervision. ART was maintained in line with current recommendations for IRIS management in PLHIV.
Anti-inflammatory therapy	Oral prednisone 6-week tapered course: 1 mg/kg/day (days 1–14), 0.5 mg/kg/day (days 15–28), and 0.25 mg/kg/day (days 29–42). Adapted from the Meintjes et al. protocol; a lower induction dose was used for cutaneous (non-CNS) disease with a low-dose taper to prevent rebound inflammation. The patient was actively monitored for steroid-related toxicities.
Oral antibacterial therapy	Oral levofloxacin 500 mg once daily plus clindamycin 300 mg three times daily initiated upon loss of IV access. Targeted isolated multidrug-resistant *E. coli* and provided broad empirical coverage for Gram-positive, anaerobic, and polymicrobial skin pathogens.
Empirical anti-herpetic therapy	Acyclovir 800 mg five times daily for 7–10 days; empirical coverage for suspected HSV/VZV co-infection. Acyclovir has no activity against MPXV.
Analgesia	Tramadol 50–100 mg every 8 h for moderate-to-severe pain, with paracetamol up to 3 g/day as an adjunct.
Local wound care	Cleansing with saline or boiled water; antisepsis with chlorhexidine 0.05% or diluted povidone-iodine (1:10). Non-adherent dressings and petroleum jelly applied to crusts. Aggressive debridement avoided.
Nutrition and supportive care	Hydration (~2 L/day), high-protein diet for tissue healing, and targeted psychosocial support to mitigate community stigma.
Daily clinical monitoring	Surveillance of temperature, pain intensity, appearance of new lesions, signs of secondary infection (purulent discharge, foul odour, expanding erythema), and ocular/mucosal involvement.
Criteria for urgent re-evaluation	Persistent fever > 38.5 °C, rapid lesion extension, dehydration, altered consciousness, or uncontrolled pain. Trigger for treatment escalation or tertiary transfer.

ART, antiretroviral therapy; HSV, herpes simplex virus; IRIS, immune reconstitution inflammatory syndrome; PLHIV, people living with HIV; VZV, varicella-zoster virus; CNS, Central Nervous System

### Response to the mpox-IRIS pragmatic management protocol

**Fig. 3:**
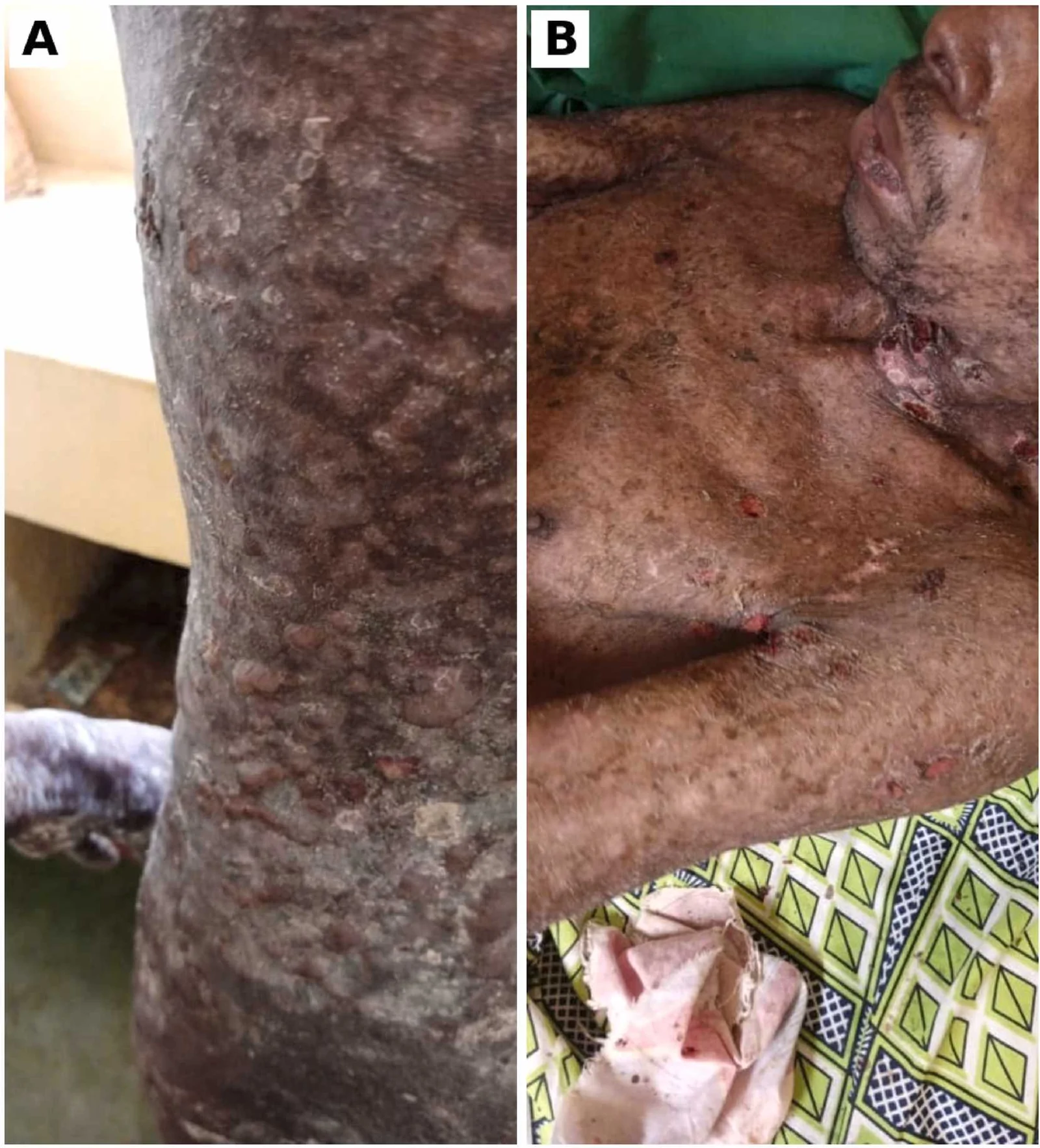
Cutaneous findings showing clinical improvement during the structured oral mpox-IRIS protocol (Table 1). (A) Right thigh showing transition of previously confluent inflammatory and hemorrhagic lesions (compare with Fig. 2) into discrete crusted papules with broad areas of re-epithelialisation and post-inflammatory hyperpigmentation. (B) Anterior chest, neck and face showing resolution of the most florid pustular phase, with residual hemorrhagic crusts at the angle of the mouth and along the chest, dried erosions and diffuse post-inflammatory hyperpigmentation. These findings developed during the six-week tapered course of oral prednisone, oral levofloxacin and clindamycin, high-dose oral acyclovir, multimodal analgesia, gentle local wound care and supportive measures, while tenofovir/lamivudine/dolutegravir was continued. The patient was eventually discharged at the family’s request after a total of 62 days of hospitalisation in an improved but still convalescent state.

Following implementation of the oral protocol, the patient experienced progressive clinical improvement over the subsequent weeks. He remained afebrile throughout the corticosteroid course, and new lesion formation ceased entirely after the first two weeks of treatment. Pre-existing confluent inflammatory and hemorrhagic plaques transitioned into discrete dry crusts with peripheral re-epithelialization and post-inflammatory hyperpigmentation on the trunk, thighs, and face (Fig. 3). Pain intensity decreased, allowing a reduction in tramadol doses, while oral intake, ambulation, and functional autonomy improved. Daily monitoring identified no criteria for urgent re-evaluation or signs of corticosteroid-related complications (such as uncontrolled superinfection, hyperglycemia, or gastrointestinal bleeding) during the 6-week taper. The remaining weeks of admission were dedicated to non-specific wound care, supervised ART, nutritional support, and rehabilitation.

### Discharge and post-discharge community outcome

After 62 days of hospitalization, the patient was discharged in an improved but convalescent state at the family's request, citing the prolonged admission, financial constraints, and social pressures. The clinical team provided detailed counseling on ART adherence, wound management under medical supervision, outpatient follow-up, and explicit avoidance of traditional remedies or irritant topical preparations on residual lesions.

Approximately 3.5 weeks post-discharge, the team was informed that the patient had died at home. Family accounts indicated that traditional topical preparations had been applied directly to his residual crusted skin lesions shortly before a rapid onset of systemic symptoms. No microbiological investigation or post-mortem examination was available to establish the exact cause of death.

## Discussion and conclusions

### Severity and clinical pattern of Clade I mpox

We describe a case of severe disseminated mpox in an adult man from eastern DRC, an area experiencing sustained clade I transmission. Multiple features align with published descriptions of severe mpox in vulnerable hosts: a high lesion count (>250) involving the face, trunk, limbs, and genitalia; polymorphic morphology; confluent necrotic plaques; secondary bacterial infection at admission; and a prolonged course requiring two months of inpatient care [3,4,8]. These clinical features meet the criteria for severe mpox as defined by the U.S. CDC [2].

### Suspected paradoxical IRIS versus natural history of clade I mpox in advanced HIV

Distinguishing paradoxical IRIS from the natural progression of Clade I mpox in advanced HIV is a major clinical challenge. Uncontrolled Clade I mpox in severely immunosuppressed hosts typically follows a relentless, non-resolving course marked by persistent high-grade fever, uninhibited viral replication, expanding ulceronecrotic lesions, and multi-organ failure culminating in septic collapse [4,6,8]. In contrast, our patient exhibited a distinct two-phase trajectory characteristic of paradoxical IRIS [9,10]: an initial phase of clinical stabilization followed by a secondary, explosive hyper-inflammatory cutaneous flare ~4 weeks after ART initiation (~2 weeks after mpox onset). Crucially, this secondary deterioration occurred while the patient was strictly afebrile, hemodynamically stable, and achieved a low plasma HIV−1 RNA level (688 copies/mL), supporting an early virologic response rather than primary ART failure or uncontrolled viral proliferation [13,14].

A central limitation in rural endemic settings is establishing an IRIS diagnosis without full immunological profiling. Due to local laboratory constraints, baseline CD4 + T-cell counts and baseline HIV RNA levels could not be obtained, preventing formal classification of "advanced HIV disease" under WHO criteria (CD4 <200 cells/µL) or direct demonstration of immune recovery. Nevertheless, clinical features—profound involuntary weight loss (admission weight: 57 kg), chronic diarrhoea, and oral candidiasis—collectively fulfilled criteria for WHO Clinical Stage 3/4 disease, strongly suggesting severe baseline cell-mediated immunosuppression [6,8]. Given the temporal sequence, low admission viral load, absence of systemic sepsis signs during deterioration, and rapid response to anti-inflammatory therapy (cessation of new pustular lesions, evolution of confluent lesions into crusts with re-epithelialisation and post-inflammatory hyperpigmentation; Fig. 3), the working diagnosis of probable mpox-associated IRIS remains the most plausible clinical framework [13,14].

### Rationale for the pragmatic oral management protocol

To our knowledge, no standardized treatment protocol for mpox-associated IRIS exists. In the absence of standardized guidelines or access to specific antivirals like tecovirimat—whose efficacy in Clade I infection was recently questioned in randomized trials [15]—management relied on a pragmatic anti-inflammatory and antimicrobial framework (Table 1) adapted to a rural center. The oral corticosteroid regimen was adapted from the Meintjes et al. trial for TB-IRIS [11], utilizing prednisone for paradoxical tuberculosis-associated IRIS (1.5 mg/kg/day for 2 weeks followed by 0.75 mg/kg/day for 2 weeks), with a slightly lower induction dose (1 mg/kg/day) appropriate for cutaneous rather than central nervous system disease, and the addition of a low-dose (0.25 mg/kg/day) intermediate step to allow a gentler taper in this very extensive skin disease. Although corticosteroids are established tools in managing severe IRIS (e.g., tuberculosis or cryptococcosis), their use in mpox remains supported only by isolated reports [13,14]. Theoretical concerns regarding enhanced viral replication or secondary sepsis warrant extreme caution and close monitoring.

**Fig. 4:**
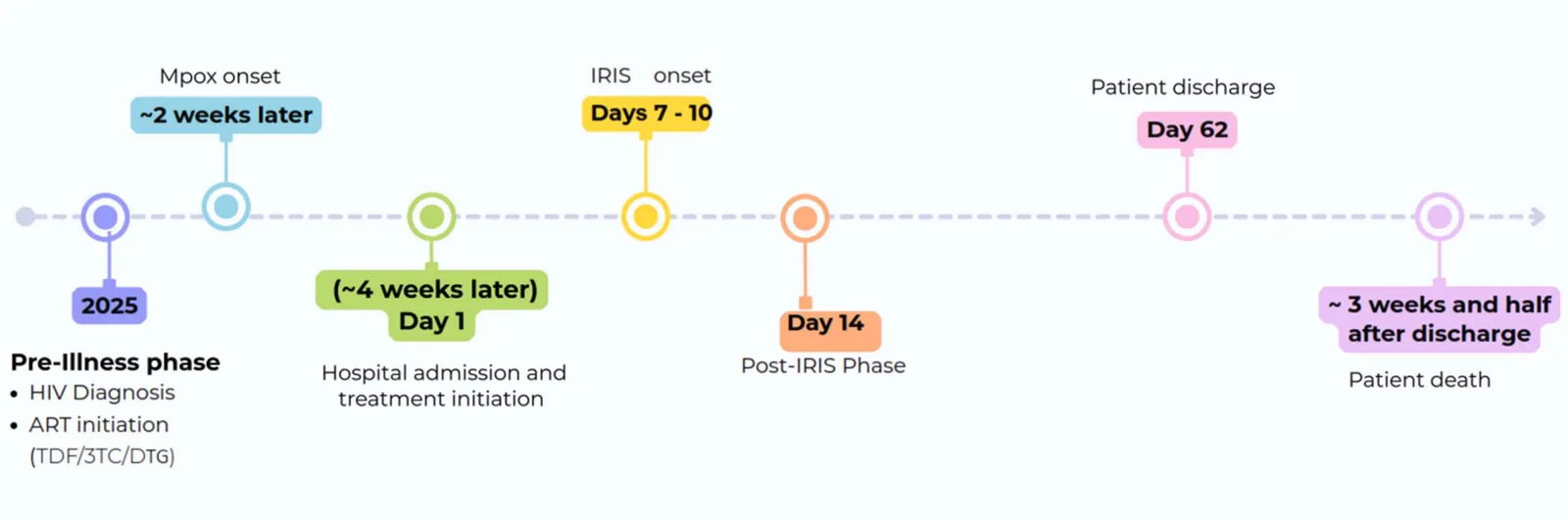
Timeline of the patient's clinical evolution. Chronological progression from HIV diagnosis and antiretroviral therapy (ART) initiation through severe mpox onset, suspected immune reconstitution inflammatory syndrome (IRIS, clinical stabilization, and the post-discharge outcome.

Antibacterial cover with levofloxacin and clindamycin was guided by purulent skin lesion cultures yielding multidrug-resistant *E. coli.* While levofloxacin targeted this Gram-negative pathogen, clindamycin was added to provide empirical coverage against common Gram-positive pathogens (including methicillin-resistant *Staphylococcus aureus*) and anaerobic organisms colonizing the extensive ulcerated skin, leveraging its documented antitoxin effects. Importantly, high-dose oral acyclovir was incorporated strictly for empirical coverage of potential co-existing HSV/VZV—which frequently cause atypical, extensive, or ulcero-necrotic anogenital lesions that clinically mimic or exacerbate severe mpox skin changes—with the explicit understanding that acyclovir possesses no antiviral activity against MPXV. Given the unavailability of specific multiplex PCR for HSV/VZV in this rural setting, empirical coverage was deemed clinically prudent (Fig. 4).

### Limitations and therapeutic gaps

Attributing recovery entirely to the anti-inflammatory protocol remains a limitation given the concurrent optimized supportive care and lack of serial CD4 + trends, follow-up viral loads, cytokine profiling, or tissue histopathology. Furthermore, managing this patient was significantly complicated by the complete unavailability of specific antiviral therapies like tecovirimat or brincidofovir at the mpox treatment center. Crucially, even when accessible, recent randomized controlled trials have questioned the clinical efficacy of tecovirimat in clade I infection [15], highlighting a profound therapeutic void that leaves clinicians in endemic, resource-limited settings entirely dependent on empirical anti-inflammatory and supportive interventions.

### Implications for clinical practice and public health

This case carries several implications. First, clinicians caring for PLHIV with newly diagnosed HIV and concurrent or recent mpox should be alert to the possibility of IRIS-associated clinical deterioration, particularly in the first weeks after ART initiation, and should not interpret clinical deterioration solely as treatment failure. Second, the in-hospital response of this patient to a pragmatic oral protocol combining a six-week tapered course of corticosteroids, oral broad-spectrum antibacterial cover, and reinforced supportive measures (Table 1) suggests that a similar approach may be considered, provided that close monitoring for corticosteroid-related complications is maintained. Third, the case underscores the urgent need for standardised diagnostic criteria for mpox-associated IRIS, modelled on those developed for tuberculosis-, cryptococcal-, and Kaposi-sarcoma-associated IRIS, and validated in prospective cohorts in endemic settings. Fourth, in the absence of robust evidence on optimal ART timing during acute mpox in PLHIV, an individualised approach balancing progressive immunosuppression against the risks of IRIS appears prudent.

### Post-discharge mortality and differential diagnosis

Determining the exact cause of death at home post-discharge (Day 62) remains limited by the absence of post-mortem or microbiological evaluation. Although initial family reports implicated unsterile traditional topical remedies applied to healing skin erosions, a broader differential diagnosis should be considered:

(i) Fulminant secondary bacterial sepsis: Application of unsterile herbal pastes onto newly re-epithelialized or eroded cutaneous barriers provides an ideal portal of entry for invasive bacterial pathogens, potentially triggering rapid septic shock in a convalescent patient.
(ii) Late sequelae and cachexia: Prolonged metabolic exhaustion, severe cutaneous scarring, post-inflammatory cachexia, and persistent immune dysregulation following a two-month hospitalization represent substantial risk factors for post-discharge mortality in severe mpox cohorts [4,8].
(iii) ART non-adherence and HIV disease progression: Discharge upon family request due to financial and social pressures may have compromised outpatient ART adherence, leading to rapid HIV virological rebound, profound CD4 collapse, and sudden clinical decline,
(iv) Unmasked opportunistic co-infections: In the absence of baseline screening, the unmasking or reactivation of an occult opportunistic co-infection post-corticosteroid taper cannot be excluded [10,12].

This tragic outcome highlights the critical importance of structured community follow-up, explicit counselling against the application of unsterile traditional remedies to healing skin, respectful and continuous engagement with traditional medicine practitioners and families, anti-stigma interventions and organised continuity-of-care strategies (including outpatient wound-care clinics and community health-worker visits) for PLHIV recovering from severe mpox in central Africa.

## Conclusions

This case reports a probable case of severe clade I mpox-associated IRIS managed in-hospital using an empirical oral corticosteroid and antimicrobial framework. While diagnostic limitations in rural endemic settings obscure definitive immune profiling, the clinical trajectory strongly supports an IRIS mechanism. The findings emphasize the need for clinicians in endemic regions to consider mpox-associated IRIS in the differential diagnosis of unexplained clinical deterioration, preventing the misidentification of immune recovery as primary ART failure. Prospective studies are required to establish standardized diagnostic criteria and evaluate target anti-inflammatory protocols. Finally, the post-discharge fatal outcome underscores that clinical recovery within a hospital facility is insufficient without cross-cultural patient education against unsterile remedies and structured community health surveillance to ensure continuity of care until complete skin healing is achieved.

## Abbreviations

ART: antiretroviral therapy; CD4^+^: cluster of differentiation 4-positive; CDC: Center for Disease Control and Prevention; CI: confidence interval; DRC: Democratic Republic of the Congo; HbA1c: glycated haemoglobin; HIV: human immunodeficiency virus; IRIS: immune reconstitution inflammatory syndrome; PCR: polymerase chain reaction; PLHIV: people living with HIV; RNA: ribonucleic acid; WHO: World Health Organization.

## CRediT authorship contribution statement

**Alain Balola Ntaboba:** Writing – review & editing, Writing – original draft, Visualization, Data curation, Conceptualization. **Christian Tshongo:** Writing – review & editing, Methodology, Investigation, Data curation. **Guillaume Ashuza Shamamba:** Writing – review & editing, Visualization, Data curation. **Victoire Urbain Hatu’m:** Writing – review & editing, Visualization, Data curation. **Alfred Kesheni Bisimwa:** Methodology, Investigation, Data curation. **Igor Akilimali Batumike:** Investigation, Data curation. **Samuel Lwamushi Makali:** Writing – review & editing, Visualization, Data curation. **David Lupande Mwenebitu:** Writing – review & editing, Visualization, Data curation. **Joanne Byrne:** Writing – review & editing, Supervision. **Virginie Gautier:** Writing – review & editing, Supervision. **Eoin R. Feeney:** Writing – review & editing, Supervision. **Jane A. O’Halloran:** Writing – review & editing, Supervision. **Patrick W.G. Mallon:** Writing – review & editing, Supervision. **Robert Colebunders:** Writing – review & editing, Supervision, Conceptualization. **Patrick D.M.C. Katoto:** Writing – review & editing, Writing – original draft, Visualization, Supervision, Investigation, Data curation, Conceptualization.

## Authors’ contributions

AKB, IAB and CTM provided clinical care to the patient during the course of his illness. ABN and PDM reviewed the medical records and drafted the manuscript. GAS, VUH, DLM, SML, JB, ERF, VG, JAO, PWM, RC and PDM critically revised the manuscript for important intellectual content. All authors read and approved the final version of the manuscript and agree to be accountable for all aspects of the work.

## Ethics declaration

Written informed consent to take part in the study and to publish the article has been obtained from all participants or their legal representatives. The privacy rights of participants have been observed.

This study included organ or tissue donors. This study includes human biological material and consent was obtained by donors, or their next of kin or legal representatives, for use in this study and for publication of the article. The samples used in this research were not sourced from executed prisoners or prisoners of conscience.

This study was performed in compliance with relevant laws, regulatory frameworks and guidelines where the research took place. This study was approved by the Comité Institutionnel d′Ethique de la Santé (CIES-UCB). (Approval No. UCB/CIES/NC/022/2024)

This research follows the CARE guidelines and the CARE checklist.

## Ethics approval and consent to participate

This case report describes the management of a single patient as part of routine clinical care at the LWIRO Mpox Treatment Center, Miti-Murhesa health zone, South Kivu, in the eastern Democratic Republic of the Congo. The relevant institutional review board/ethics committee was informed, and approval for the publication of de-identified clinical information and images was obtained from the Université Catholique de Bukavu (*UCB/CIES/NC/022/2024*).

## Consent for publication

Written informed consent for publication of clinical details and accompanying images was obtained from the patient prior to deterioration of his condition. A copy of the consent form is available for review by the Editor-in-Chief of this journal upon request. Given the fatal outcome and the impossibility of obtaining additional consent post-mortem, the existing prospectively obtained consent has also been confirmed in writing by the patient’s next of kin.

## Funding

No specific funding was received for the preparation of this case report. SMART (*NCT05745987*) and PREGMPOX (*NCT06442501*) projects have supported clinical care.

## Declaration of Competing Interest

The authors declare that they have no known competing financial interests or personal relationships that could have appeared to influence the work reported in this paper.

## Data Availability

All data relevant to this case report are included in the manuscript and its figures. Additional de-identified clinical data are available from the corresponding author on reasonable request.
